# SapC-DOPS nanovesicles induce Smac- and Bax-dependent apoptosis through mitochondrial activation in neuroblastomas

**DOI:** 10.1186/s12943-015-0336-y

**Published:** 2015-04-08

**Authors:** Mahaboob K Sulaiman, Zhengtao Chu, Victor M Blanco, Subrahmanya D Vallabhapurapu, Robert S Franco, Xiaoyang Qi

**Affiliations:** Division of Hematology and Oncology, Department of Internal Medicine, University of Cincinnati College of Medicine, Cincinnati, Ohio USA; Divison of Human Genetics, Department of Pediatrics, Cincinnati Children’s Hospital Medical Center, Cincinnati, Ohio USA

**Keywords:** SapC-DOPS, Saposin C, Dioleoylphosphatidylserine, Mitochondria-mediated apoptosis, Smac/Diablo, Bax polymerization

## Abstract

**Background:**

High toxicity, morbidity and secondary malignancy render chemotherapy of neuroblastoma inefficient, prompting the search for novel compounds. Nanovesicles offer great promise in imaging and treatment of cancer. SapC-DOPS, a stable nanovesicle formed from the lysosomal protein saposin C and dioleoylphosphatidylserine possess strong affinity for abundantly exposed surface phosphatidylserine on cancer cells. Here, we show that SapC-DOPS effectively targets and suppresses neuroblastoma growth and elucidate the molecular mechanism of SapC-DOPS action in neuroblastoma *in vitro*.

**Methods:**

*In vivo* targeting of neuroblastoma was assessed in xenograft mice injected intravenously with fluorescently-labeled SapC-DOPS. Xenografted tumors were also used to demonstrate its therapeutic efficacy. Apoptosis induction *in vivo* was evaluated in tumor sections using the TUNEL assay. The mechanisms underlying the induction of apoptosis by SapC-DOPS were addressed through measurements of cell viability, mitochondrial membrane potential (ΔΨM), flow cytometric DNA fragmentation assays and by immunoblot analysis of second mitochondria-derived activator of caspases (Smac), Bax, Cytochrome c (Cyto c) and Caspase-3 in the cytosol or in mitochondrial fractions of cultured neuroblastoma cells.

**Results:**

SapC-DOPS showed specific targeting and prevented the growth of human neuroblastoma xenografts in mice. In neuroblastoma cells *in vitro*, apoptosis occurred via a series of steps that included: (1) loss of ΔΨM and increased mitochondrial superoxide formation; (2) cytosolic release of Smac, Cyto c, AIF; and (3) mitochondrial translocation and polymerization of Bax. ShRNA-mediated Smac knockdown and V5 peptide-mediated Bax inhibition decreased cytosolic Smac and Cyto c release along with caspase activation and abrogated apoptosis, indicating that Smac and Bax are critical mediators of SapC-DOPS action. Similarly, pretreatment with the mitochondria-stabilizing agent bongkrekic acid decreased apoptosis indicating that loss of ΔΨM is critical for SapC-DOPS activity. Apoptosis induction was not critically dependent on reactive oxygen species (ROS) production and Cyclophilin D, since pretreatment with N-acetyl cysteine and cyclosporine A, respectively, did not prevent Smac or Cyto c release.

**Conclusions:**

Taken together, our results indicate that SapC-DOPS acts through a mitochondria-mediated pathway accompanied by an early release of Smac and Bax. Specific tumor-targeting capacity and anticancer efficacy of SapC-DOPS supports its potential as a dual imaging and therapeutic agent in neuroblastoma therapy.

**Electronic supplementary material:**

The online version of this article (doi:10.1186/s12943-015-0336-y) contains supplementary material, which is available to authorized users.

## Introduction

Nanotechnology is transforming cancer therapy by improving drug delivery, imaging and selective targeting of tumor cells [1-3]. In the past, several attempts have been made to formulate nanovesicles from components normally present in cells [4]. In this direction, SapC-DOPS, a nanovesicle made from saposin C and dioleylphosphatidylserine, is a unique protein-lipid complex that selectively targets and kills human cancer cells *in vitro* and *in vivo* [1,5-12]*.* Saposin C is an 80 kDa heat-stable, protease-resistant protein containing distinct functional domains; that functions as a co-activator of sphingolipid-degrading lysosomal hydrolases (sphingomyelinase and acid β-glucosidase) [13]. Owing to the presence of a fusogenic domain comprising two amphipathic α − helices containing four Lys residues (K13, K17, K26, and K38), SapC exhibits natural affinity towards negatively charged phospholipids such as phosphatidylserine (PS). This interaction occurs at low pH (pKa of 5.3) and is critical for SapC activation [13,14]. We have previously assembled SapC and DOPS into stable nanovesicles and its efficacy and safety profiles have been established in various forms of cancer [1,5,6,8,12]. SapC-DOPS is hypothesized to bind to exposed PS on the cancer cell surface and induce apoptosis by increasing intracellular ceramide level leading to subsequent caspase activation [8]. However, the precise intracellular pathway(s) mediating SapC-DOPS induced apoptotic cancer cell death is still unknown.

Neuroblastoma accounts for 15% of all pediatric cancer mortalities and is the most common extracranial tumor in young adults [15]. Aggressive chemotherapy and radiation protocols have failed to improve the survival rates significantly in children with high-risk disease [16]. Efficacy of chemotherapy in neuroblastoma is less than satisfactory due to several factors such as high toxicity, severe morbidity and risk of secondary malignancy [17]. Moreover, in patients with relapse the long-term survival rates are < 50% emphasizing the need for novel, non-genotoxic targeted therapies. Elevation of intracellular ceramide, a known regulator of mitochondrial function, was noticed during *in vitro* treatment of neuroblastoma cell lines with SapC-DOPS [8]. Ceramide induces apoptosis via the mitochondrial pathway [18]. Mitochondria play a central role in the induction of apoptosis by acting as both a major amplification step and the principal site of action for pro- and anti-apoptotic members of the Bcl-2 family [19]. Whereas the anti-apoptotic members (e.g.,Bcl-2 and Bcl-xL) confine apoptogenic proteins within the mitochondrial intermembrane space by promoting pore closure, the pro-apoptotic proteins (e.g., Bax, Bak and Bid) that translocate from the cytosol to mitochondria promote pore opening [20]. In addition, it is known that these pro-apoptotic molecules promote pore formation independently or in combination with other mitochondrial proteins such as the voltage-dependent anion channel (VDAC) effecting the release of apoptogenic proteins such as Cyto c, Smac/Diablo and apoptosis inducing factor (AIF) from the intermembrane space [21-23]. Final commitment to apoptosis is postulated to occur by the following sequence of events: formation of pores or channels in the outer mitochondrial membrane, opening of pores, loss of mitochondrial membrane potential (ΔΨM), apoptogenic protein release from mitochondria and caspase activation [24]. In this report we evaluate the *in vivo* targeting and antitumor capacity of SapC-DOPS in mice bearing neuroblastoma xenografts, and address the molecular mechanisms underlying neuroblastoma cell death after SapC-DOPS exposure. Based on past observations, we test the hypothesis that SapC-DOPS-induced apoptotic cell death is caused by mitochondrial dysfunction, apoptogenic protein release and caspase activation. Cell viability, mitochondrial function and redistribution of apoptotic proteins are assessed *in vitro* in the neuroblastoma cell lines SK-N-SH and IMR-32, representative of non-metastatic and metastatic neuroblastoma, respectively. The *in vivo* efficacy of SapC-DOPS in suppressing neuroblastoma growth and the insights obtained into its mechanism of action support its potential as a novel therapeutic agent for the treatment of neuroblastoma.

## Materials and methods

### Reagents and antibodies

The following reagents and antibodies were used: Bongkrekic acid (Santa Cruz Biotechnology, La Jolla, CA), JC-1 (eBioscience, San Diego, CA), Cyclosporine A, 2′,7′-dichlorofluorescein acetate (DCFH-DA), N-acetyl cysteine (Sigma, St.Louis, MO), Dioleylphosphatidylserine (Avanti Lipids, Alabaster, AL), 3-(4,5-dimethylthiazol-2-yl)-2,5-diphenyltetrazolium bromide (MTT) ((Roche Diagnostics, Indianapolis, IN), and disuccinyl suberate (Thermo Scientific Fischer, Rockford, IL). Anti-Bcl-2, anti-β-Actin (Abcam, Cambridge, MA), anti-Cyto c (eBioscience, San Diego, CA), anti-AIF, anti-caspase-3, anti-cleaved caspase-3 (Cell Signaling Technology, Boston, MA, anti-Survivin, Smac/Diablo, α-Tubulin (Novus biological, Littleton, CO), anti-COX-4, anti-Bax (N-20; Santa Cruz Biotechnology, La Jolla, CA), anti-Bax (polymer-recognizing A67 clone; Sigma, St.Louis, MO) and anti- cleaved PARP (Millipore, Bedford, MA).

Animal maintenance and experimental procedures were carried out in accordance with the US National Institute of health Guidelines for Use of Experimental Animals and approved by the Institutional Animal Care and Use Committee of the University of Cincinnati and Cincinnati Children's Hospital Medical Center.

### Preparation and characterization of SapC-DOPS nanovesicles

The procedures for production of SapC-DOPS nanovesicles have been described in detail before [1,6-9]. Briefly, recombinantly expressed and purified saposin C along with solvent-dried dioleylphosphatidylserine were mixed in acidic citrate-phosphate buffer and freshly assembled into nanovesicles by bath sonication. The lipophilic, infrared dye CellVue Maroon (CVM) was added during SapC-DOPS assembly to fluorescently label the nanovesicles for *in vivo* imaging of neuroblastoma human xenografts. The nanovesicles are stable for at least a week, when stored at 4°C. TEM analysis for surface morphology was performed as described earlier [25].

### Mouse xenografts and cell culture

Human neuroblastoma CHLA-20 was a gift from Thomas Inge (Cincinnati Children’s Hospital Medical Center); the origin and culture conditions were previously described [26]. Athymic nude mice (nu/nu, NIH) (15 mice per group), were injected with 7.5 × 10^6^ cells subcutaneously to initiate tumor growth. When tumors reached a volume of 400 mm^3^, five doses of either SapC-DOPS (SapC 4 mg/kg body weight, DOPS 2 mg/kg body weight) or PBS (control) were intratumorally administered once every 3 days. Tumor growth was assessed periodically with a caliper, and after 16 days, tumors were excised, weighted, and processed for hematoxylin and eosin staining and apoptosis (TUNEL) assays.

The human neuroblastoma SK-N-SH and IMR-32 cell lines were obtained from American Type Culture Collection and grown in AMEM supplemented with 10% FBS. Human Schwann cells (ScienCell Research Laboratories, Carlsbad, CA) were grown as recommended by the supplier. After overnight attachment, cells were treated with either DOPS or SapC-DOPS for concentration- or time-dependence assays. Where indicated, cells were pretreated for 60 min with bongkrekic acid, cyclosporine A or N-acetyl cysteine.

### Cell viability and apoptosis assays

Cell viability was assessed with a standard assay using the tetrazolium dye MTT (3-(4,5-dimethylthiazol-2-yl)-2,5-diphenyltetrazolium bromide) as previously described [6] three days after initiating treatment. The following methods were employed to assess SapC-DOPS-induced cell death: G6PD release assay (Life Technologies, Grand Island, NY), was performed according to the manufacturer’s instructions, DAPI staining was performed as described earlier [27], caspase activation through cleaved caspase-3 and cleaved PARP fragments was evaluated by Western blotting, and cell cycle analysis was performed on a FACS Calibur (Becton Dickinson) in serum-starved, synchronized cells after fixation with 80% ethanol at −20°C for 20 min followed by staining with 100 μg/ml of RNase and 25 μg/ml of PI [28]. Cell cycle phase was analyzed with the CellQuest-Pro software program (Becton Dickinson). *In vivo* apoptosis was measured by TUNEL staining as described earlier [8].

### Evaluation of mitochondrial membrane potential (ΔΨM) and ROS production

Following treatment with SapC-DOPS, cells in triplicate were washed with PBS and evaluated for concentration- and time-dependent changes in ΔΨM by resuspension in fresh JC-1 containing medium, followed by 30 min incubation in the dark at room temperature [28]. Fluorescence intensity was measured with excitation at 490 nm and the emission monitored at 530 (monomer) and 590 (aggregate) nm, using a BMG microplate reader (BMG Labtech, Inc., Durham, NC). The ratio between green and red fluorescence provides an estimate of ΔΨM that is independent of the mitochondrial mass. For the ROS assay, cells treated with SapC-DOPS were exposed to DCFH-DA for 15 min at 37°C. Fluorescence excitation and emission wavelengths were set at 480 and 530 nm respectively, using a BMG microplate reader (BMG Labtech, Inc., Durham, NC). Mitochondrial superoxide was detected using the fluorescent Mito-Sox probe (Invitrogen). Cells were incubated in Hank’s buffer with 2 μM MitoSox-Red for 30 min at 37°C in a 5% CO_2_ atmosphere, washed with PBS and the fluorescence assessed by flow cytometry. Positive control cells were pretreated with 20 μM Antimycin A for 20 min at room temperature. We used the FL1, FL2 and FL3 channels of a FACScalibur flow cytometer (15 mW argon ion laser tuned at 488 nm; CellQuest software, Becton Dickinson Biosciences). Thresholds were adjusted by using non-stained and stained cells for MitoSox-Red fluorescence.

### Flow cytometric evaluation of Ca^2+^ by Fluo-3 AM assay

Intracellular calcium was measured by flow cytometry using the cell permeant, Ca^2+^-sensitive fluorescent dye Fluo-3 AM (Life technologies, Carlsbad, CA) [29]. After treatment with SapC-DOPS for different time points, cells were washed in serum-free Advanced-MEM media, and incubated with Fluo-3 AM for 30 min at 37°C. Later, cells were washed with HEPES (4-(2-hydroxyethyl)-1-piperazineethanesulfonic acid) buffer, trypsinized and centrifuged at 3,500 rpm for 5 min. The pellet was resuspended in HEPES buffer and analyzed using FACS Calibur (Becton Dickinson) with excitation at 488 nm and emission at 525 nm. Approximately 10,000 events were counted.

### Western blotting

After treatment with SapC-DOPS, cells were trypsinized and lysed with RIPA buffer (Sigma) containing protease inhibitor (Thermo Pierce) for 30 min on ice. The lysates were centrifuged at 11,300 g for 20 min at 4°C to collect supernatant. Protein concentration was determined by the BCA method (Thermo Fischer Scientific, Rockford, IL). Equal amounts of proteins (30–50 μg) were separated by 4-20% gradient sodium dodecyl sulphate polyacrylamide gel electrophoresis (SDS-PAGE) and transferred to PVDF membrane (Amersham). After blocking, the membrane was incubated overnight at 4°C with primary antibodies. Following this, blots were probed with the appropriate Li-COR secondary antibodies conjugated with IRDye 800 CW or IRDye 680 LT. Proteins were visualized using an Odyssey IR scanner and quantified using Odyssey software (LI-COR Biosciences, Lincoln, NE).

### Mitochondrial and cytosolic fractionation

The mitochondrial and cytoplasmic fractions were separated using the Mitochondrial/Cytosol fractionation kit (BioVision, CA, USA) as per the manufacturer’s instructions. Briefly, whole-cell pellets dissolved in cytosolic fraction extraction buffer were subjected to 55 strokes in a 2 ml Dounce homogenizer on ice. The homogenate was centrifuged at 3,500 rpm for 10 min at 4°C to pellet nuclei and unbroken cells. The supernatant was subsequently centrifuged at 13,000 rpm for 30 min at 4°C to obtain cytosolic supernatant and the mitochondrial pellet. Mitochondrial pellets were resuspended in mitochondrial extraction buffer by gentle vortex for 30 sec.

### Bax oligomerization and bax inhibition

Bax oligomerization with cross linking was detected as described previously [27]. Briefly, cells after treatment with SapC-DOPS cells were washed in conjugating buffer followed by cross linking with 2 mM disuccinyl suberate in non-reducing buffer and incubated for 30 min at room temperature. Reaction was quenched by addition of Tris–HCl (pH 7.5) and incubation at room temperature for 15 min. The samples were then solubilized in lysis buffer containing Nonidet P-40 without a reducing agent and centrifuged at 12,000 x g for 10 min. Bax oligomers were detected using the A67 clone that exclusively detects the polymerized form. Bax inhibition was performed as described earlier [30]. Briefly, cells were pre-incubated with Bax inhibiting peptide (V5) or negative control peptide (EMD Millipore,Chicago, IL) for one hour, prior to SapC-DOPS treatment.

### Lentiviral infection and stable knockdown of Smac/Diablo

Permanent knockdown of Smac was achieved by expression of pre-validated shRNA targeting the sequence CCGACAATATACAAGTTTACT in Smac (shSmac) available as clone ID TRCN04513 in the Sigma-TRC consortium database. DNA oligonucleotides encoding for shSmac were annealed and cloned into pLKO.1 puro. Lentivirus particles carrying shSmac were produced by transfecting 293T cells with pLKO.1-puro-shSmac together with viral packaging vectors (psPAX2, pMD2G) by calcium phosphate transfection at the Cincinnati Children’s Hospital Medical Center viral vector core facility. Three days post infection of SK-N-SH cells with Smac shRNA containing virus, cells were selected in a medium containing puromycin (Life Technologies, Grand Island, NY). Efficiency of the knockdown was checked by Western blot. Cells transfected with the empty vector served as control.

### Statistical analysis

Data are represented as the mean ± SE. Statistical analyses were done with the Student’s t test and P < 0.05 was considered significant.

## Results

### SapC-DOPS targets neuroblastoma and inhibits tumor growth *in vivo*

**Figure 1:**
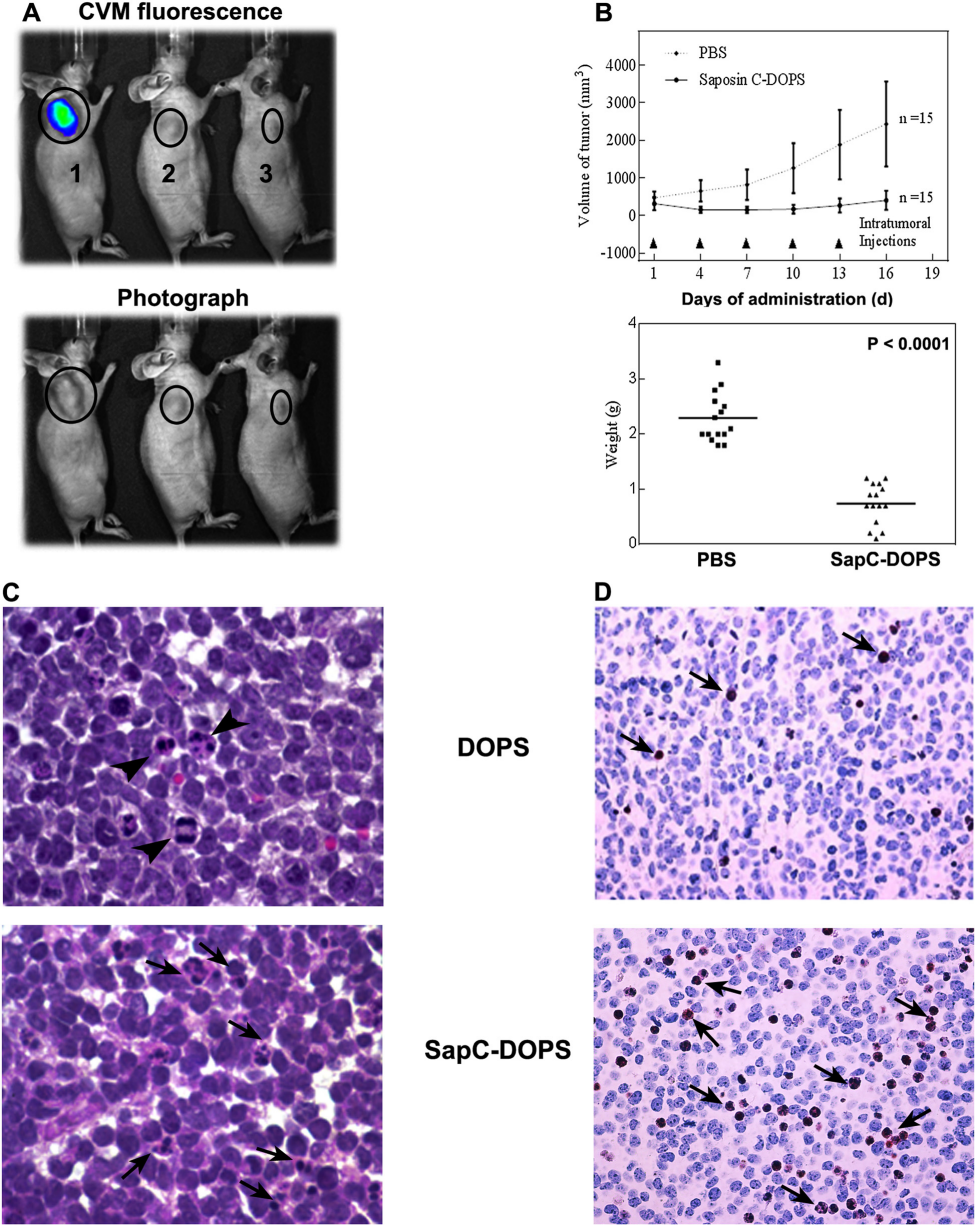
**Preparation and tumor-targeting potential of SapC-DOPS in mice xenografts. A)** Treatment of athymic nude mice bearing neuroblastoma (CHLA-20) xenografts with CVM-labeled SapC-DOPS (1), CVM-DOPS (2) or SapC-CVM (3). Animals were imaged 24 h after injection with exposure time of 1 s. Saposin C, 4.2 mg/kg; dioleoylphosphatidylserine, 2 mg/kg; CellVue Maroon, 6 μmol. **B)** Evaluation of tumor burden in SapC-DOPS treated mice xenografts. Mice xenografts (n = 15) were treated with five intratumoral injections of SapC-DOPS (SapC 4 mg/kg, DOPS 2 mg/kg) or PBS every 3 days and followed for tumor growth. **C)** Histological examination of the neuroblastoma tumor tissue in mice after intratumoral injection of DOPS and SapC-DOPS. Original magnification: 1000x. **D)** Evaluation of apoptosis by TUNEL staining (arrows) in DOPS- and SapC-DOPS treated tumors. Original magnification: 40x.

Saposin C and dioleoylphosphatidylserine were assembled (pH 5.0) at a molar ratio of 1:7 for *in vivo* studies or 1:3 for *in vitro* studies. Tumor targeting was evaluated by real-time fluorescent imaging, following an intravenous injection of fluorescently (CVM)-labeled SapC-DOPS nanovesicles (mouse 1), CVM-labeled DOPS (mouse 2), or SapC + CVM (mouse 3) in nude mice bearing human neuroblastoma xenografts produced by flank injections of CHLA-20 cells. At 24 h post injection, fluorescent signal was only detected in tumor-bearing mouse that received CVM-labeled SapC-DOPS (Figure 1A).

To assess the therapeutic efficacy of SapC-DOPS on neuroblastoma, mice bearing CHLA-20 neuroblastoma xenografts were subjected to intratumoral injections of SapC-DOPS (SapC = 4 mg/kg, DOPS = 2 mg/kg) or phosphate buffered saline (PBS). Mice treated with SapC-DOPS showed a significant inhibition of tumor growth (P = 0.0097) and decreased tumor weight (P < 0.0001) when compared to mice treated with PBS (Figure 1B). Histological evaluation by hematoxylin-eosin (H&E) and terminal deoxynucleotidyltransferase dUTP nick end labeling (TUNEL) staining showed extensive induction of apoptosis within neuroblastoma tissue (Figure 1C, D) in the mouse xenografts. Since SapC-DOPS showed specific tumor targeting and induced apoptosis in mouse xenografts, we proceeded to determine the mechanism behind apoptosis induction in established neuroblastoma cell lines.

### SapC-DOPS triggers apoptosis of neuroblastoma cells

**Figure 2:**
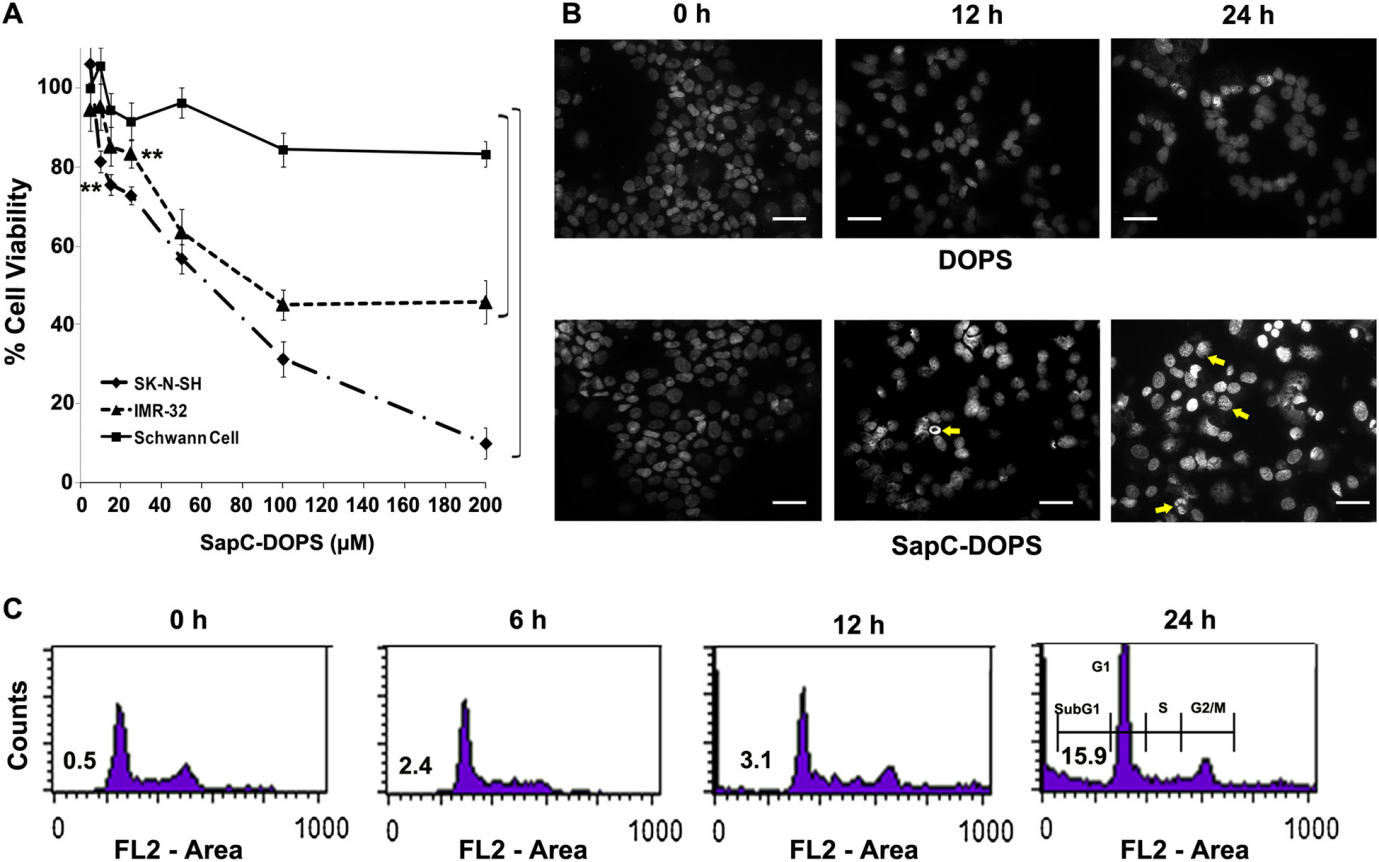
**SapC-DOPS induces apoptosis in neuroblastoma cell lines. A)** Viability of SK-N-SH, IMR-32 and human Schwann cells measured by the MTT assay. **represents the lowest concentration from which P value is significant i.e., < 0.001. **B)** Microscopic images of SK-N-SH cells after treatment with 350 μM DOPS or 50 μM SapC-DOPS. Arrows indicate morphological changes such as cell shrinkage and chromatin condensation. Original magnification: 20x). **C)** Changes in cell cycle following treatment of SK-N-SH cells with 50 μM SapC-DOPS for 24 h. The value represents percentage of cells in Sub-G1 region. *P < 0.05, **P < 0.001 when compared to control. Points, mean of three to five independent experiments; bars, SE.

We hypothesized that the characteristically acidic tumor microenvironment facilitates the binding of SapC-DOPS to exposed PS on tumor cells, activates lysosomal glucosylceramide breakdown, and elevates ceramide levels leading to apoptotic cell death [6]. Although elevated levels of sphinganine, sphingosine and ceramides i.e. molecules capable of regulating mitochondrial function were reported earlier [8] the role of SapC-DOPS in mitochondrial function was not critically evaluated. Viability assays in cultured cells measured after 72 h, showed that SapC-DOPS induced significant cell death in a dose-dependent manner at a concentration of 15 μM or above in SK-N-SH and IMR-32 human neuroblastoma cells, but not in normal, human Schwann cells [*P* < 0.001; Figure 2A]. In contrast, the maximum loss of viability in Schwann cells when exposed to the highest concentration of 200 μM SapC-DOPS was only around 10-15%. IMR-32 cells proved to be more resistant to SapC-DOPS than SK-N-SH cells, in as much as 200 μM dose did not induce further cell death, after a 3 day culture period. The microscopic changes observed were characteristic of apoptosis and included irregular shapes, nuclear chromatin condensation and cell shrinkage [Figure 2B, see arrows]. Treatment with up to 350 μM DOPS alone did not elicit cell death [Additional file 1: Figure S1A]. Likewise, SK-N-SH and IMR-32 cells treated with SapC did not show any significant decrease in viability (data not shown). Cell cycle analysis by flow cytometry showed substantial DNA fragmentation as indicated by the time-dependent increase in sub-G_0_/G1 events [Figure 2C]. A G6PD release assay, used to estimate necrotic cell death, yielded negative results (P = 0.46; n = 5; Additional file 1: Figure S1B) and further confirmed that apoptosis is the predominant mechanism underlying SapC-DOPS-induced cell death.

### SapC-DOPS disrupts mitochondrial membrane potential in neuroblastoma cells

**Figure 3:**
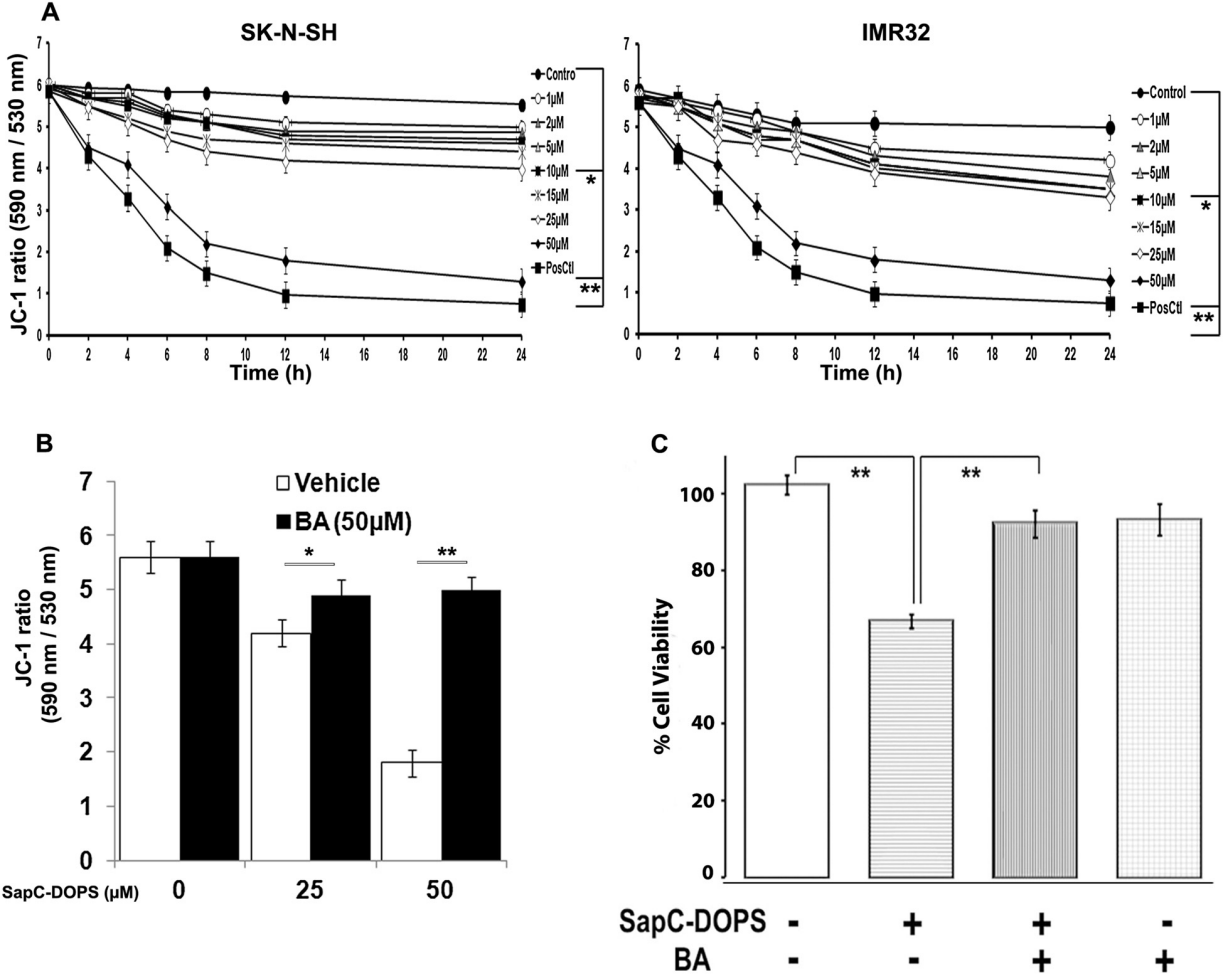
**SapC-DOPS induces loss of mitochondrial potential (ΔΨM). A)** Dose- and time-dependent changes in ΔΨM evaluated by JC-1 ratio of red and green fluorescence following SapC-DOPS treatment in SK-N-SH and IMR-32 cells. **B)** ΔΨM measured by JC-1 ratio in bongkrekic acid-pretreated SK-N-SH cells following SapC-DOPS treatment for 24 h. **C)** Viability measured by MTT assay in SK-N-SH cells following pre-treatment with bongkrekic acid (BA) and subsequent treatment with 50 μM SapC-DOPS for 72 h. *P < 0.05, **P < 0.001 when compared to control. Points, mean of three to five independent experiments; bars, SE.

Disruption of ΔΨM, which indicates mitochondrial dysfunction and loss of membrane integrity, is considered an early step in apoptosis [31,32]. In healthy cells, the potentiometric mitochondrial dye JC-1 preferentially accumulates within the mitochondria and spontaneously aggregates into a polymer emitting red fluorescence. In apoptotic, unhealthy cells with low potential, JC-1 remains in monomeric form emitting green fluorescence. Treatment of neuroblastoma cells with SapC-DOPS resulted in pronounced loss of ΔΨM in both dose- and time-dependent manners [Figure 3A]. The decrease in red/green fluorescence ratio of JC-1 was significant (*P* < 0.001) after 4 h at a dose of 50 μM SapC-DOPS, with a more pronounced effect at later times. At 24 h, 5 μM was sufficient to significantly induce loss in ΔΨM (*P* < 0.01). Pretreatment with 50 μM BA, a ΔΨM stabilizer [33], prevented loss of ΔΨM [Figure 3C], and significantly attenuated SapC-DOPS induced cell death in SK-N-SH cells [*P* < 0.001; Figure 3D]. These observations suggest that a loss of ΔΨM underlies the induction of neuroblastoma apoptosis by SapC-DOPS.

### SapC-DOPS induces translocation of apoptogenic proteins and oligomerization

**Figure 4:**
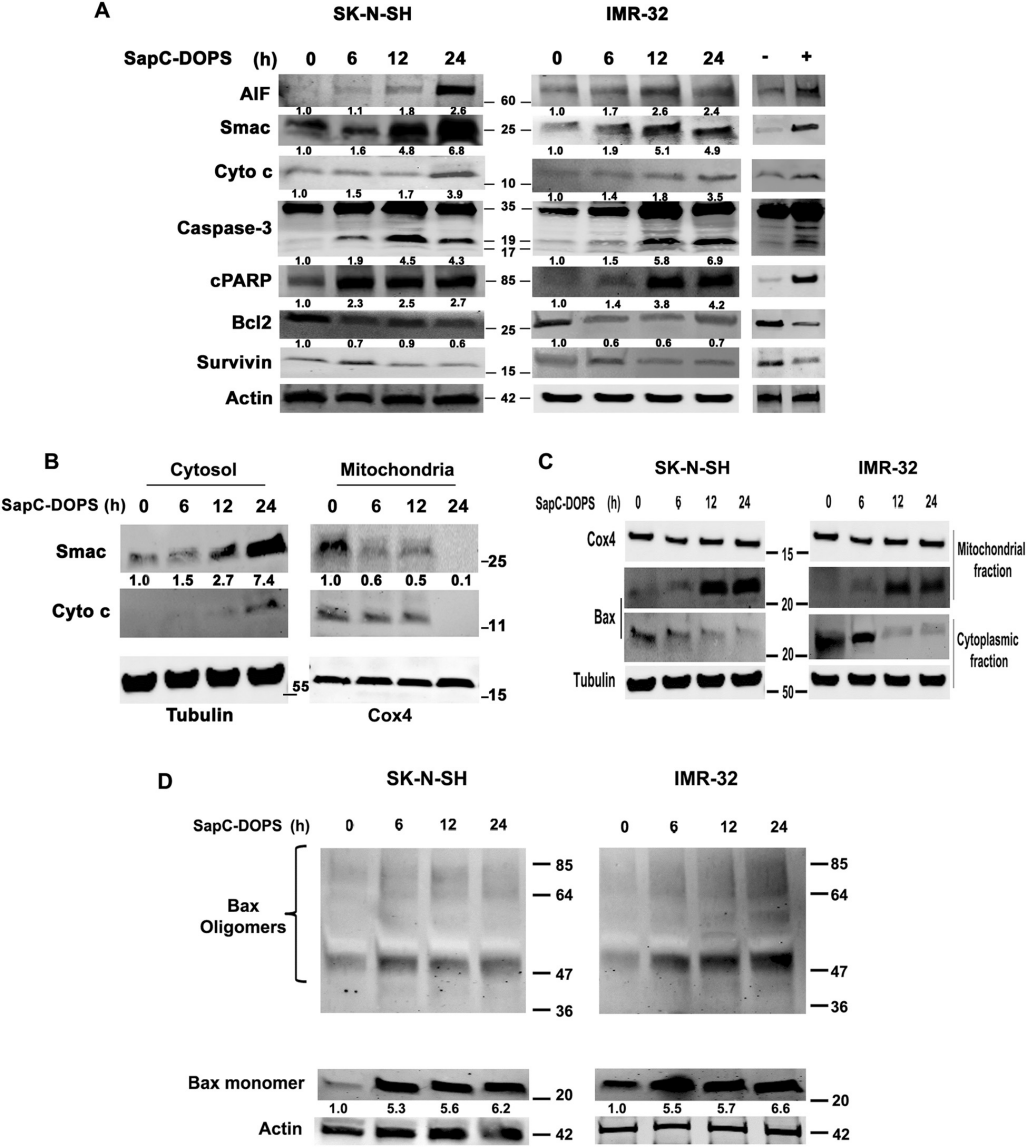
**SapC-DOPS treatment causes redistribution of apoptogenic proteins and Bax oligomerization in mitochondria of neuroblastoma cells. A)** Immunoblots from whole cell extracts showing apoptotic protein expression changes following treatment with 50 μM SapC-DOPS. Fractions indicate fold-change estimated by densitometric analysis of proteins normalized to β-Actin corresponding to the lane. Fold change indicated for caspase-3 corresponds to the cleaved 19 kDa fragment. Right lanes: (−) refers to negative control (Schwann cells treated with SapC-DOPS) and (+) refers to positive control (SK-N-SH cells treated with 10 μM staurosporine) for 24 h. **B)** Relocation of Smac and Cyto c in SK-N-SH cells. Cox4 and Tubulin served as loading control for the mitochondrial and cytoplasmic fractions, respectively. **C)** Bax redistribution in neuroblastoma cells following SapC-DOPS treatment. **D)** Bax oligomerization in neuroblastoma cells. **P < 0.001. Points, mean of three to five experiments; bars, SE. Western blots are representative of three independent experiments.

During mitochondria-mediated apoptosis the outer mitochondrial membrane becomes permeable, a process that is necessary for apoptogenic protein release and caspase activation [34]. Smac, Cyto c, and AIF are proteins released to cytosol from mitochondria in response to death stimuli [35]; Smac and Cyto c cause apoptosis by caspase-dependent pathways whereas AIF works by caspase-independent pathways [36]. As shown in Figure 4A, treatment of SK-N-SH and IMR-32 cells with SapC-DOPS caused an increase in the expression of AIF, Smac and Cyto c. These changes were paralleled by a marked increase in the active caspase-3 fragment, an early execution phase signal in human neuroblastoma [37], as well as in cleaved poly-ADP-ribose polymerase (cPARP), a substrate of caspase-3. Partial decreases in the expression levels of anti-apoptotic protein Bcl-2 were also observed, particularly in IMR-32 cells. Survivin, a member of the XIAP family of anti-apoptotic proteins, showed a transient increase at 6 h but returned near baseline levels at 24 h post-treatment in both cell lines. To determine whether SapC-DOPS causes a redistribution of Smac and Cyto c to the cytosol, mitochondrial as well as cytosolic levels of both proteins were estimated simultaneously by Western blotting. These results showed that Smac release preceded that of Cyto c (Figure 4B). Control experiments performed in SK-N-SH cells showed that DOPS alone did not alter the expression levels of apoptotic proteins [Additional file 2: Figure S2A]. Likewise, no change in apoptotic protein expression was noticed upon treatment of SK-N-SH and IMR-32 cells with SapC alone (data not shown). These results demonstrate that SapC-DOPS treatment increases AIF, Smac and Cyto c protein levels, triggers a cellular redistribution of Smac and Cyto c, and induces cPARP formation and activation of caspase-3.

### Bax activation is necessary for SapC-DOPS-induced apoptosis

Bax, a crucial proapoptotic factor of the Bcl-2 family localizes to the cytosol but translocates to the mitochondria in response to various apoptotic stimuli [38]. Relocated Bax molecules facilitate mitochondrial release of Smac and/or Cyto c to the cytosol by forming channels on the outer mitochondrial membrane via homo- or hetero-dimerization with members of the permeability transition pore (PTP) such as the voltage-dependent anionic channel (VDAC), the adenine nucleotide translocator, and cyclophylin D, among others[39]. In untreated SK-N-SH and IMR-32 cells, Bax proteins were predominantly found in the cytosolic fraction of [Figure 4C]. SapC-DOPS treatment induced Bax translocation from the cytosolic to the mitochondrial compartment [Figure 4C]. Bax polymerization, detected with a monoclonal antibody (6A7) that specifically recognizes Bax oligomers [27], was confirmed in whole cell extracts of SK-N-SH and IMR-32 cells [Figure 4D]. A sustained increase in total Bax monomer expression was detected 6 h after treatment in both cell lines.

### Relocation of Smac and Cyto c is independent of ROS formation, Cyclophilin D channel activity and increased Ca^2+^ levels

**Figure 5:**
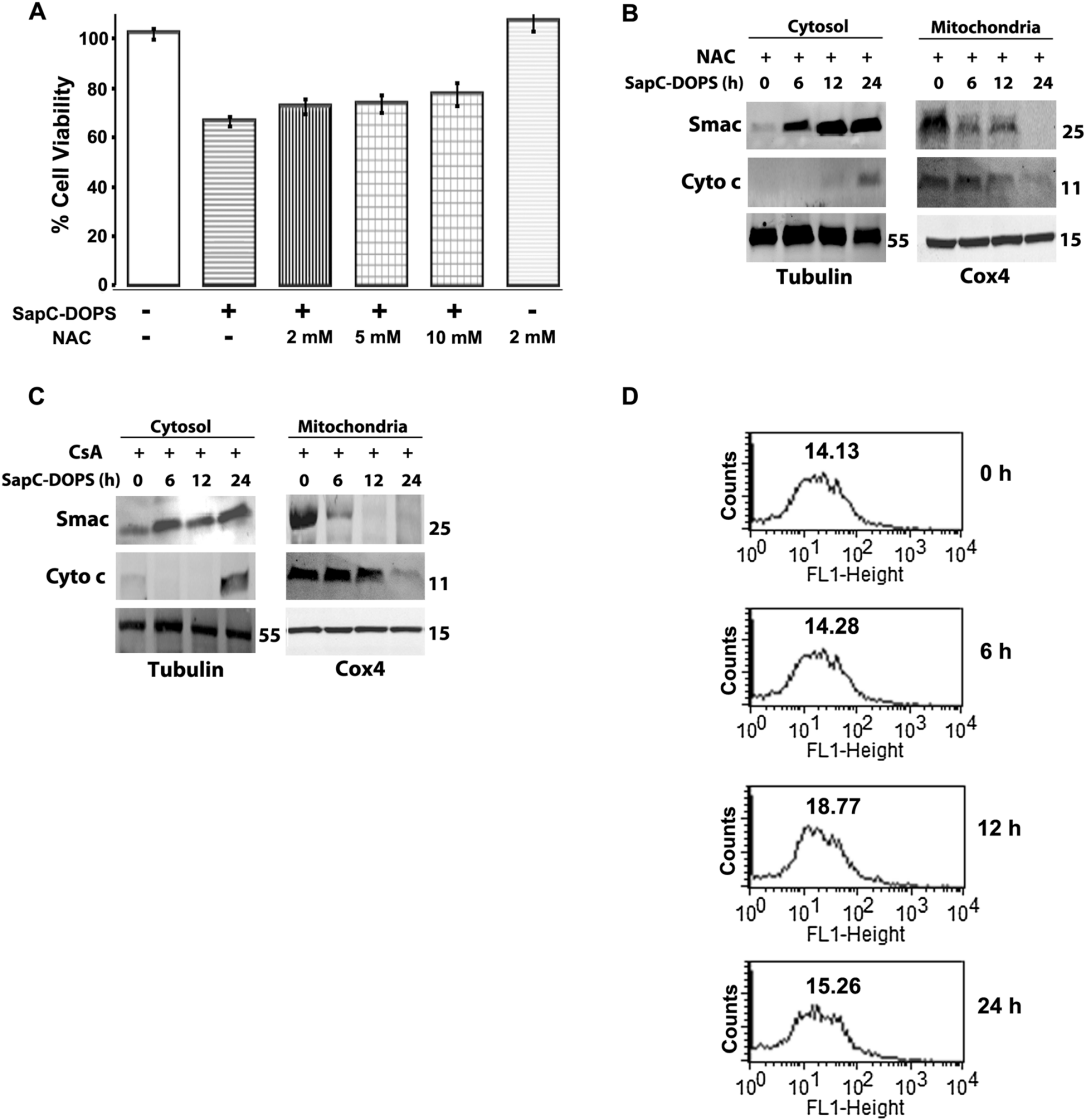
**Apoptotic redistribution of Smac and Cyto c is independent of ROS formation, Cyclophilin D activity and Ca** ^**2+**^ **. A)** Effect of N-acetyl cysteine (NAC)-mediated ROS inhibition on viability of SK-N-SH cells following SapC-DOPS treatment. **B)** Effect of pretreatment with 2 mM N-acetyl cysteine (NAC) following SapC-DOPS (50 μM) treatment on Smac and Cyto c relocation in SK-N-SH cells. **C)** Effect of 1 μM cyclosporine A (CsA)-pretreatment on Smac and Cyto c relocation following SapC-DOPS (50 μM) treatment in SK-N-SH cells. **D)** Flow cytometric measurement of Ca^2+^ using Fluo-3 AM in SK-N-SH cells treated with 50 μM SapC-DOPS. Values represent geometric mean of fluorescence. Points, mean of three to five experiments; bars, SE. Western blots are representative of three independent experiments.

Loss of ΔΨM results in several deleterious intracellular outcomes such as generation of ROS that promotes oxidative stress and the subsequent release of mitochondrial proteins Smac, Cyto c and AIF to the cytosol. Since generation of ROS has been shown to accelerate cell death in neuroblastoma cells [40], we examined the effects of SapC-DOPS on intracellular ROS formation. SapC-DOPS treatment induced significant mitochondrial superoxide formation, as shown by an increase in MitoSox-red fluorescence intensity, 24 h post-treatment, in both SK-N-SH and IMR-32 cells [Additional file 1: Figure S1C and S1D]. The next set of experiments was performed to differentiate whether apoptotic induction by SapC-DOPS is driven by ROS or the intrinsic pathway. First, we blocked ROS formation by pre-treating SK-N-SH cells with 2 mM NAC and evaluated Smac and Cyto c relocation. Pretreatment with NAC neither rescued cell viability (Figure 5A) nor prevented Smac and Cyto c relocation from mitochondria to cytosol (Figure 5B). These results suggest that induction of ROS does not mediate the apoptotic effects of SapC-DOPS and may be a secondary consequence of SapC-DOPS action.

The permeability transition pore (PTP), an incompletely characterized channel complex that may include Bax, is a major determinant of the mitochondrial release of Smac, Cyto c and AIF during apoptosis [41]. To determine the possible role of the PTP in SapC-DOPS mediated mobilization of Smac and Cyto c, SK-N-SH cells were pre-incubated with 1 μM cyclosporine A, which blocks PTP opening by binding to cyclophilin D [42] As shown in Figure 5C, PTP inhibition did not alter the time-dependent relocation of Cyto c and Smac from mitochondria to the cytosol. Elevation of intracellular Ca^2+^ is an important trigger for PTP opening and the subsequent release of apoptogenic proteins. Flow cytometric analysis with the Ca^2+^-sensitive dye Fluo-3 AM showed no changes in intracellular Ca^2+^ levels following SapC-DOPS treatment [Figure 5D]. These results show that SapC-DOPS induces mitochondrial Smac and Cyto c release without mediation by Cyclophilin D/PTP or Ca^2+^.

### Smac and Bax play critical roles in SapC-DOPS induced apoptosis

**Figure 6:**
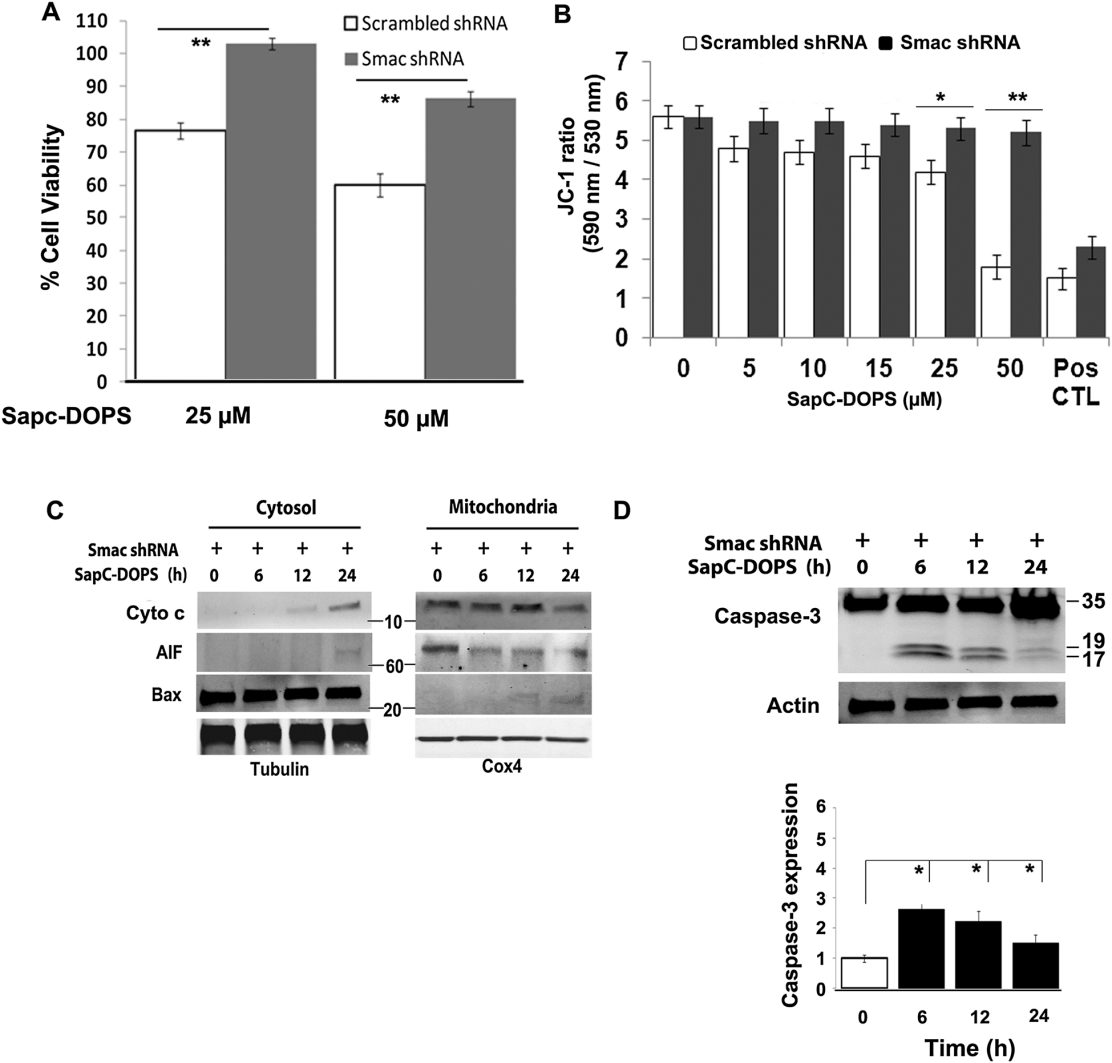
**Smac plays an important role in SapC-DOPS-induced apoptosis. A)** SK-N-SH cell viability assessed by MTT assay after treatment with 50 nM control scrambled shRNA (empty bars) or 50 nM Smac shRNA (filled bars) and subsequent SapC-DOPS treatment **B)** Evaluation of ΔΨM by JC-1 assay in Smac-knockdown SK-N-SH cells following 50 μM SapC-DOPS treatment for 24 h. Pos CTL refers to treatment with 50 μM 2-[2-(3-Chlorophenyl)hydrazinylyidene]propanedinitrile (CCCP). **C)** Redistribution of apoptogenic proteins following 50 μM SapC-DOPS treatment for 24 h in Smac-knockdown SK-N-SH cells. **(D)** Caspase-3 activation in SapC-DOPS (50 μM) treated Smac-knockdown SK-N-SH cells. Densitometry graph shows relative changes in cleaved caspase-3 fragment expression normalized to β-Actin. *P < 0.05, **P < 0.001. Points, mean of three to five experiments; bars, SE. Western blots are representative of three independent experiments.

Studies have shown that Smac/Diablo protein release can occur independently of Cyto c release from the mitochondria during apoptosis [22,23]. As noted above, Smac release occurred by 6 h, the earliest time point in our study that coincided with caspase-3 and PARP cleavage. Therefore, to assess the relevance of Smac in SapC-DOPS-induced apoptosis, we performed shRNA-mediated Smac knockdown in SK-N-SH cells. Cells were transfected with 50 nM lentiviral Smac shRNA *in vitro*, and viable cell number assessed over time. Whole cell immunoblots confirmed significantly decreased Smac protein levels, as measured 48 h after transfection, with selective protein knockdown of >96% [Additional file 2: Figure S2B]. Apoptosis-related off-target effects were ruled out by comparing levels of Cyto c in control and shSmac-treated cells. Knocking down Smac expression significantly decreased SapC-DOPS-induced apoptosis as denoted by reduced cell death (*P* < 0.001; Figure 6A), and prevented the ΔΨM loss, reflected by increased JC-1 red/green fluorescence ratio (Figure 6B). While Smac knockdown did not affect mitochondrial release of AIF and Cyto c, the translocation of Bax to mitochondria was abrogated [Figure 6C] and the expression of cleaved caspase-3 fragments was significantly attenuated in SapC-DOPS-treated SK-N-SH cells [*P* < 0.01; Figure 6D]. Taken together, these findings suggest that Smac plays a critical functional role in SapC-DOPS-induced apoptosis.

**Figure 7:**
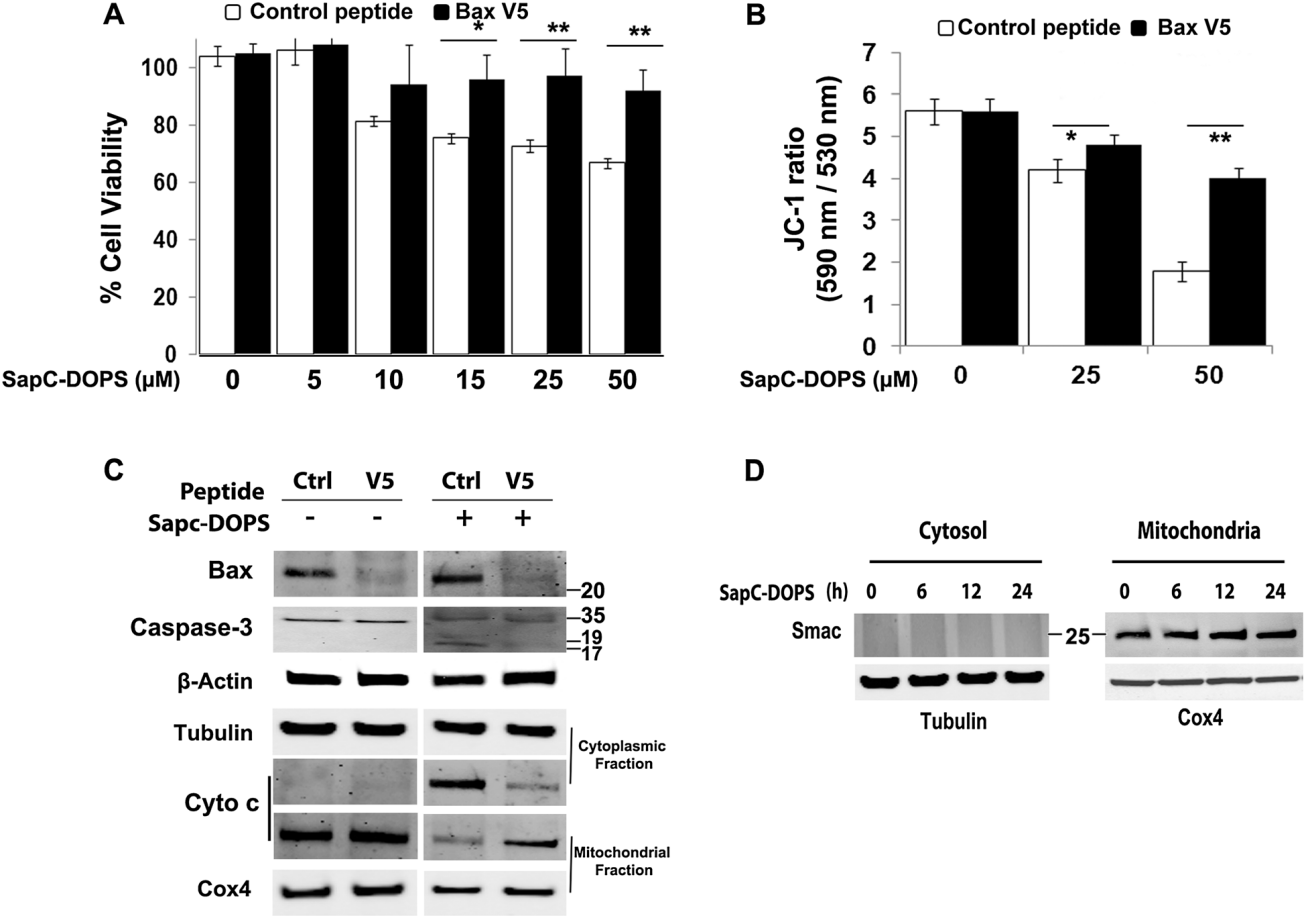
**Bax inhibition reduces SapC-DOPS-induced apoptosis. A)** MTT assay in control peptide-treated (empty bar) and V5 peptide-treated (Bax-inhibited; filled bars) SK-N-SH cells following SapC-DOPS treatment. **B)** ΔΨM measured by JC-1 assay in SK-N-SH cells following SapC-DOPS treatment for 24 h. **C)** Apoptotic protein expression in control-peptide and Bax-V5-peptide pre-treated SK-N-SH cells following 50 μM SapC-DOPS treatment for 24 h. **D)** Changes in Smac expression in cytosolic and mitochondrial extracts of Bax-inhibited SK-N-SH cells treated with 50 μM SapC-DOPS. *P < 0.05, **P < 0.001. Points, mean of three to five experiments; bars, SE. Western blots are representative of three independent experiments.

Because SapC-DOPS induces Bax translocation and oligomerization, we questioned whether Bax is necessary for its pro-apoptotic action. SK-N-SH cells were treated with the Bax-inhibitory peptide V5 (50 μM) or a control peptide (50 μM) for 30 min followed by treatment with 50 μM SapC-DOPS. Suggesting an important role for Bax in the mitochondrial depolarization caused by SapC-DOPS, V5 pretreatment led to a significant reduction in cell death [*P* < 0.001; Figure 7A] and attenuation of ΔΨM loss [Figure 7B]. Consistent with decreased apoptosis, Bax inhibition also attenuated Smac and Cyto c mitochondrial release and reduced caspase-3 activation [Figure 7C, 7D]. Pretreatment with the control peptide neither inhibited Bax nor decreased SapC-DOPS-induced apoptosis. These results indicate that Bax is a critical player during SapC-DOPS induced apoptosis.

In summary, these findings strongly suggest that SapC-DOPS preferentially induces apoptosis in neuroblastoma by a mitochondrial-mediated pathway.

## Discussion

This study shows that SapC-DOPS, an antitumor agent formed by the naturally-occurring protein Saposin C and DOPS, targets neuroblastoma cells and inhibits neuroblastoma growth *in vitro* and *in vivo*. Previous work from our lab has shown that the preferential targeting of SapC-DOPS nanovesicles to cancer cells, while sparing normal ones, is due to higher levels of exposed phosphatidylserine on their outer membranes [1,6-10]. Upon cell binding, SapC-DOPS is internalized and SapC activates lysosomal hydrolases that degrade glucosylceramide and sphingomyelin, resulting in the accumulation of ceramide [8], a well-known apoptosis inducer [18]. In the present study we perform a detailed analysis of SapC-DOPS actions in two neuroblastoma cell lines, and reveal that tumor toxicity results from mitochondrial-mediated apoptosis triggered by disrupted ΔΨM, mitochondrial release of Cyto c and Smac, Bax relocation and oligomerization, and activation of Caspase 3. With the efficacy of SapC-DOPS having been confirmed in numerous solid tumor models [1,6,8-10], the elucidation of SapC-DOPS mode of action is of critical importance to design clinical trials, predict clinical outcomes as well as anticipate and manage potential adverse effects.

Our *in vitro* results show that SapC-DOPS exerts dose-dependent cytotoxicity in IMR-32 and SK-N-SH neuroblastoma cells, but have little effect on the viability of normal Schwann cells. Treated neuroblastoma cell cultures showed microscopy features typical of apoptosis, including cell shrinkage and chromatin condensation, while flow cytometric analysis of DNA content showed progressive DNA fragmentation. On the other hand, necrotic cell death was ruled out, as evidenced by a G6PD assay. Next, we addressed the molecular bases of SapC-DOPS induced apoptosis, by first evaluating possible changes in ΔΨM. Mitochondria maintain ΔΨM by controlling ion transport via channels residing in the inner and outer mitochondrial membranes. Loss of ΔΨM is an early requirement of apoptosis [43] and precedes chromatin condensation [44]. Upon induction of apoptosis, a series of events induce mitochondrial outer membrane permeabilization (MOMP), altering ΔΨM. Under certain conditions loss of ΔΨM acts as an initiator, whereas in others it follows its onset [45]. MOMP is mainly controlled by the Bcl-2 family of proteins that either reside on the mitochondrial membrane, or reassemble there after translocation from cytoplasm. Upon oligomerization, they form new channels that release mitochondrial apoptogenic proteins like Cyto c, Smac and AIF. We show here that SapC-DOPS nanovesicles induce, within 6 h, a significant decrease in ΔΨM, which is paralleled by a decrease in mitochondrial Smac and increased cPARP and caspase 3 cleavage, denoting apoptotic cell death. Loss of ΔΨM is critical for SapC-DOPS tumor toxicity, as cell viability was significantly rescued following pre-treatment of cells with the ΔΨM stabilizer agent BA. Further experiments showed that SapC-DOPS induced a delayed release (by 24 h) of other important pro-apoptotic proteins, namely Cyto c and AIF, as well as translocation of Bax from cytosol to mitochondria with oligomerization of Bax monomers.

The nature and specificity of mitochondrial protein channels and their individual preferences towards the apoptogenic proteins Smac and Cyto c is under intense debate [46]. However, accumulating evidence suggests that several channels function independently or in conjunction with the Bcl-2 family to determine the internal milieu of the organelle [41,47]. The phenomenon termed “mitochondrial permeability transition” (MPT) reflects the opening of the mitochondrial permeability transition pore (PTP), triggering an influx of water into the mitochondrial matrix due to osmosis and resulting in MOMP, which promotes apoptotic caspase-dependent and –independent cell death. VDAC, ANT, Cyclophilin D and the Translocator protein (18 kD) are putative constituents of the PTP [47,48], although knockout experiments have shown VDAC and ANT to be dispensable for MPT-driven MOMP, suggesting that alternate channels may regulate cell death [48]. Pre-treatment with cyclosporine A, which inhibits the PTP by binding to Cyclophilin D, failed to prevent SapC-DOPS-induced mitochondrial efflux of Smac or Cyto c. Mitochondrial Ca^2+^ overload is a critical activator of the PTP [49]. In this study, however, flow cytometry with the Ca^2+^-sensitive dye Fluo-3 AM showed that total intracellular Ca^2+^ levels were not significantly altered after SapC-DOPS treatment. Collectively these results indicate that neither Cyclophilin D-mediated PTP opening nor intracellular Ca^2+^ elevations are critical for SapC-DOPS-induced apoptosis of neuroblastoma cells.

Bax oligomerization is proposed to form megachannels in the outer mitochondrial membrane facilitating pro-apoptotic protein release [36]. In support of an essential role of Bax, we observed that mitochondrial Bax translocation is rapid following SapC-DOPS treatment, and is necessary for Smac and Cyto c release from the mitochondria. However, significant oligomerization of Bax is only noticed at 24 h, which coincides with the peak of Smac and Cyto c release from the mitochondria. Although many studies suggest that in the absence of Bax no cytosolic Smac release occurs [50], there are reports which show that cytotoxins [51] as well as apoptosis inducers such as AT-101 [27] directly target mitochondria and trigger Smac release irrespective of Bax activation. In the present study, Bax inhibition led to complete loss of cytosolic Smac release and an attenuated apoptosis as seen by diminished Cyto c release and caspase-3 activation. These results indicate that Bax is required for SapC-DOPS induced apoptosis. However, the trigger for Bax polymerization is currently unknown. ROS formation may trigger Bax conformational change and translocation [52]. Consistent with this, we observed an increase in ROS formation by 6 h after SapC-DOPS treatment. However, generation of ROS is not required for apoptosis induction since pretreatment with ROS scavenger, N-acetyl cysteine (NAC) failed to prevent SapC-DOPS-induced cytosolic Smac and Cyto c release. Therefore, our results imply that SapC-DOPS-induced ROS formation is not critical for apoptosis, but may enhance Bax oligomerization to form megachannels. The relationship between Smac release and Bax activation is complex and is still under active investigation. Early mitochondrial release pointed to a crucial role for Smac in SapC-DOPS induced apoptosis. It has been reported that initial Smac release requires active caspases [53], and we observed caspase activation corresponding to this time point. In general, during apoptosis the temporal release of Cyto c from mitochondria precedes Smac release [54]. However, some anticancer drugs selectively release Smac rather than Cyto c [55]. In agreement with the latter, we noticed selective cytosolic Smac release at the early stages of SapC-DOPS-induced apoptosis, whereas Cyto c release was not evident until 24 h. A marked increase in cell viability, retention of ΔΨM and reduction in SapC-DOPS induced apoptosis as seen by a decrease in cytosolic AIF and Cyto c release in Smac knockout cells further confirmed the essential role of Smac. These results are consistent with other reports that show Smac as an essential pro-apoptotic molecule that determines anticancer activity [56].

The two human neuroblastoma cell lines we used in the present study have been shown to differ in their expression of MYCN protein, which plays an important role in the synergistic activation of Smac in some neuroblastoma cells [57]. However, we observed that SapC-DOPS induced mitochondrial Smac and Cyto c release pattern was similar in the two neuroblastoma cell lines examined, despite different MYCN status. Further studies may be needed to clarify if SapC-DOPS-induced Bax oligomerization culminates in Smac release independently, or in combination with other Bcl-2 family members.

In summary, our present findings indicate that SapC-DOPS shows selective *in vivo* tumor targeting and exert significant tumor inhibition in mice bearing human neuroblastoma xenografts. Apoptosis was observed *in vivo* and *in vitro* and occurred through a mitochondrial pathway as demonstrated by a loss of mitochondrial ΔΨM and increased mitochondrial superoxide formation, with mitochondrial release of apoptogenic proteins such as AIF, Smac and Cyto c, and mitochondrial translocation and polymerization of cytosolic Bax. Gene knockdown and inhibition studies established Smac and Bax as major regulators of SapC-DOPS-induced apoptosis of neuroblastoma cells. These results, and the benign safety profile evidenced by several studies, suggest that SapC-DOPS may provide an effective therapeutic approach against neuroblastoma.

## Additional files

Additional file 1: Figure S1. Evaluation of necrosis and mitochondrial superoxide formation. **A)** MTT assay of SK-N-SH cells treated with DOPS. **B)** Necrosis measured by G6PD release in SK-N-SH cells following treatment with SapC, DOPS and SapC-DOPS for 24 h. **C)** Mean MitoSox-Red fluorescence following 50 μM SapC-DOPS treatment of neuroblastoma cells for 24 h. Pos CTL stands for pre-treatment with 20 μM Antimycin A. **D)** Quantification of fold changes in MitoSox intensity after treatment with 50 μM SapC, 350 μM DOPS or SapC-DOPS (25, 50 μM) for 24 h. Additional file 2: Figure S2. Protein expression analysis in SK-N-SH cells. **A)** Expression of apoptotic proteins following treatment with 350 μM DOPS. Fractions indicate fold-change estimated by densitometric analysis of proteins normalized to β-Actin corresponding to the lane. **B)** ShRNA-mediated knockdown of Smac in SK-N-SH cells. Percentages represent reduction in Smac normalized to β-Actin.
