# Supplementary figures and images for: SapC-DOPS nanovesicles induce Smac- and Bax-dependent apoptosis through mitochondrial activation in neuroblastomas
Source: Mol Cancer. 2015 Apr 8;14:78. doi: 10.1186/s12943-015-0336-y (PMC4397704; doi:10.1186/s12943-015-0336-y)

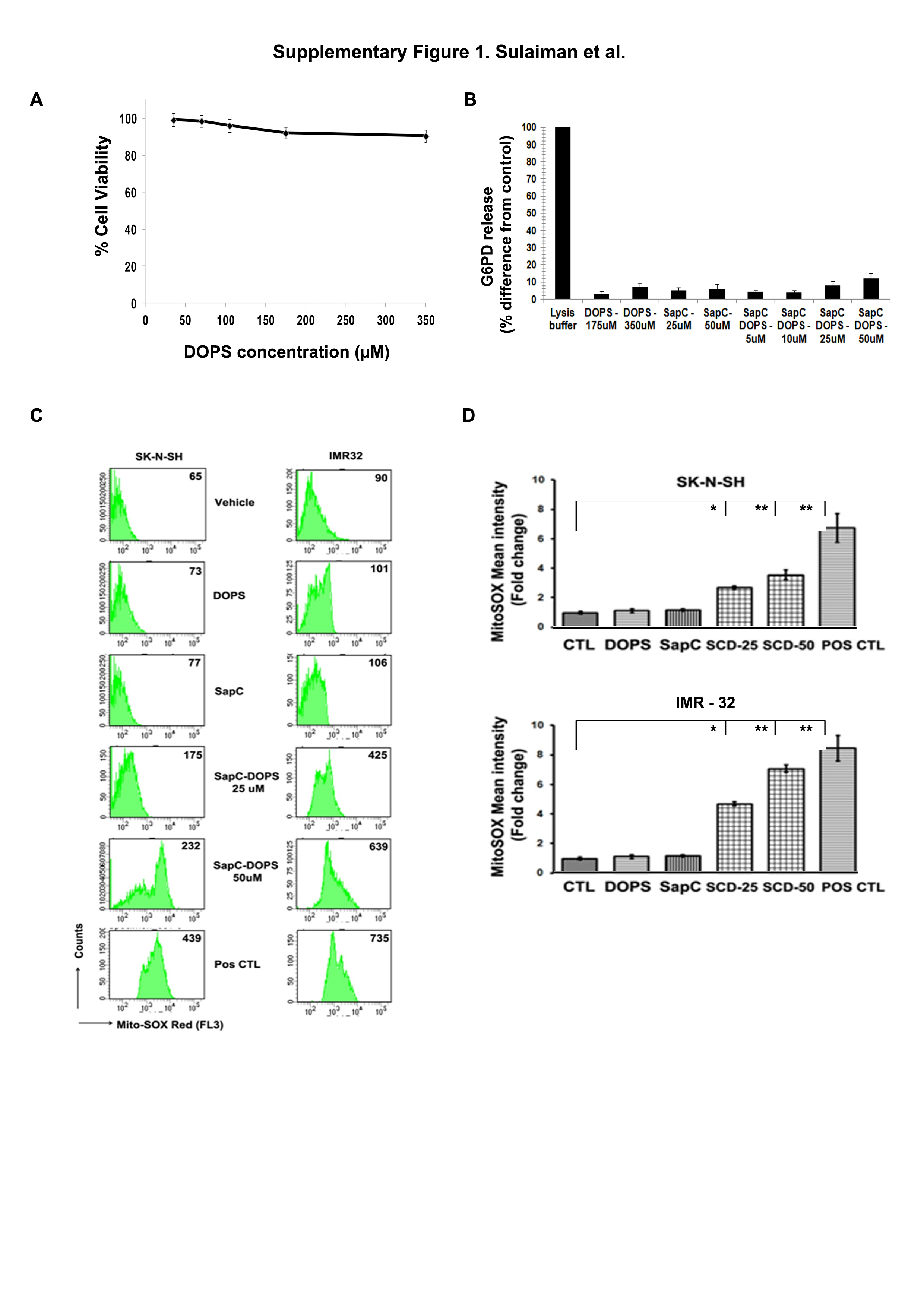

Supplement: Additional file 1: Figure S1. — Evaluation of necrosis and mitochondrial superoxide formation. A) MTT assay of SK-N-SH cells treated with DOPS. B) Necrosis measured by G6PD release in SK-N-SH cells following treatment with SapC, DOPS and SapC-DOPS for 24 h. C) Mean MitoSox-Red fluorescence following 50 μM SapC-DOPS treatment of neuroblastoma cells for 24 h. Pos CTL stands for pre-treatment with 20 μM Antimycin A. D) Quantification of fold changes in MitoSox intensity after treatment with 50 μM SapC, 350 μM DOPS or SapC-DOPS (25, 50 μM) for 24 h. [file 12943_2015_336_MOESM1_ESM.tiff]

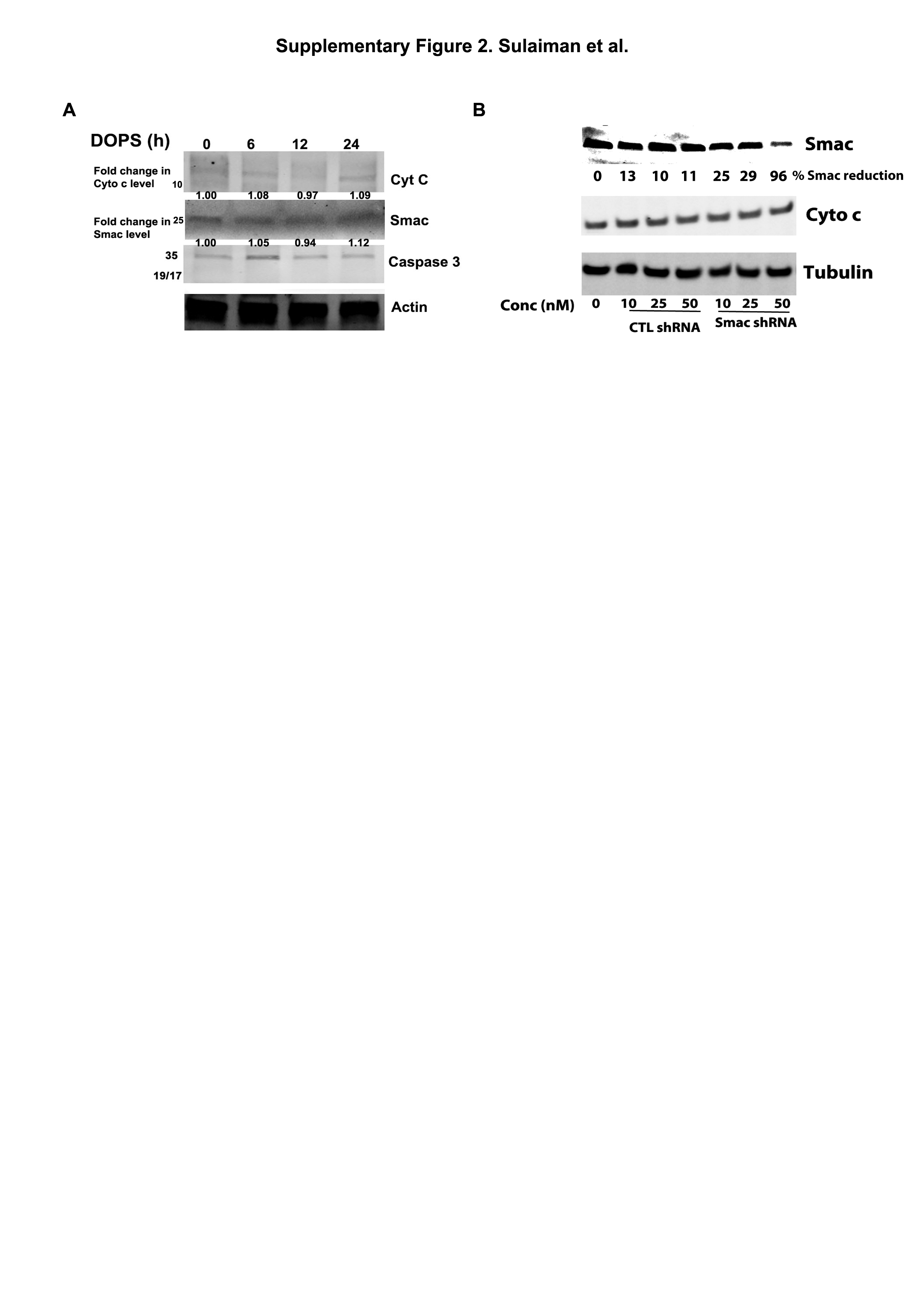

Supplement: Additional file 2: Figure S2. — Protein expression analysis in SK-N-SH cells. A) Expression of apoptotic proteins following treatment with 350 μM DOPS. Fractions indicate fold-change estimated by densitometric analysis of proteins normalized to β-Actin corresponding to the lane. B) ShRNA-mediated knockdown of Smac in SK-N-SH cells. Percentages represent reduction in Smac normalized to β-Actin. [file 12943_2015_336_MOESM2_ESM.tiff]
